# Ginger extract promotes pancreatic islets regeneration in streptozotocin-induced diabetic rats

**DOI:** 10.1042/BSR20241510

**Published:** 2025-03-13

**Authors:** Manal S. Abbood, Amani M. Al-Adsani, Suzanne A. Al-Bustan

**Affiliations:** 1 Department of Biological Sciences, Faculty of Science, Kuwait University, Shadadiyah, Kuwait P.O. Box 5969, Safat 13060, Kuwait

**Keywords:** diabetes, β-cell development, insulin, gene expression, ginger extract, regeneration

## Abstract

Ginger (*Zingiber officinale*) exerts an antidiabetic effect by restoring pancreatic β-cells. The present study aimed to investigate the mechanism by which ginger extract induces the regeneration of functional β-cells in diabetic rats. Sprague–Dawley rats (*n*=27) were divided into three groups: normal rats given double distilled water (ddH_2_O) (NC, *n*=11), diabetic rats (injected with 60 mg/kg streptozotocin) given ddH_2_O (DC, *n*=8), and diabetic rats treated with aqueous ginger extract (DG, *n*=8). The effect of ginger extract intake on the differential expression of neurogenin-3 (*Neurog3*), V-maf musculoaponeurotic fibrosarcoma oncogene homolog B (*Mafb*), insulin 2 (*Ins2),* and glucagon (*Gcg*) was assessed using quantitative real-time PCR after one and eight weeks of treatment. The pancreatic insulin source was determined using immunohistochemical analysis. After one week, ginger treatment significantly up-regulated the expression of both *Neurog3* and *Mafb* in the DG rats compared with the DC rats. However, after eight weeks, the mRNA levels of these genes dropped significantly in parallel with the up-regulation of *Ins2* and *Gcg* expression, resulting in increased serum insulin levels, weight, and lowered fasting blood glucose levels. Immunohistochemical analysis revealed a restored β-cell mass and islet architecture in the DG group. Ginger extract exerts an antidiabetic effect by acting on pancreatic progenitors and α-cells to restore β-cell mass in streptozotocin-induced diabetic rats. These findings suggest that ginger extract could be a potential stimulator of β-cell neogenesis, which provides an alternative to meet the increasing demand for exogenous insulin in patients with diabetes.

## Introduction

Diabetes mellitus is the most widespread endocrine disorder that imposes a major burden on the healthcare system. The worldwide prevalence of diabetes mellitus is projected to increase from 463 million in 2019 to 700 million cases in 2045 [1]. The lack of insulin-secreting β-cells is the major hallmark of diabetes leading to glucose intolerance and sustained hyperglycemia [2]. Currently, exogenous insulin is administered to support most patients with diabetes to overcome the absence of functional pancreatic β-cells [3]. However, this approach focuses on managing diabetes rather than resolving the underlying causes and is associated with several side effects, including hypoglycemia [4]. Therefore, the regeneration of β-cell mass could be the appropriate therapy for diabetes [5]. However, a detailed understanding of the triggering factors and processes of β-cell development and regeneration is elusive.

Extensive research has uncovered fundamental transcription factors underlying the orchestration of pancreatic endocrinogenesis [6]. During pancreatic organogenesis, all endocrine lineages, including β-cells, arise from neurogenin-3 (NEUROG3)+ endocrine progenitors. *Neurog3* encodes for a basic helix-loop-helix transcription factor essential for the formation of endocrine lineages. However, it is not co-expressed with any endocrine hormones in adult pancreatic cells [7]. In developing β-cells, NEUROG3 triggers the expression of essential downstream transcription factors involved in β-cell differentiation, including paired box 4 (*Pax4*) [8], which exhibits a mutually exclusive expression pattern with aristaless-related homeobox (*Arx*), favoring the differentiation of α- and polypeptide (PP)-cell lineages [9].

Unlike other transcription factors, large musculoaponeurotic fibrosarcoma (MAF) family proteins are expressed at later developmental stages, primarily in hormone-producing cells [10]. MAFB was detected earlier than MAFA in both insulin (INS)+ and glucagon (GCG)+ cells at embryonic day (E) 10.5. However, its expression is progressively restricted to α-cells postnatally [11]. In contrast, MAFA is expressed only in β-cells at E13.5 and completely replaces MAFB in adult β-cells [12]. In developing β-cells, MAFB directly regulates the transcription of *Mafa*, as well as that of other genes critical for β-cell functions, such as *Ins1/2*, pancreatic and duodenal homeobox 1 (*Pdx1),* and solute carrier family 2 member 2 (*Slc2a2*) [11,13]. Knockout studies revealed reduced numbers of both cell types with delayed β-cell formation in *Mafb* null mice, suggesting that *Mafb* is required for the terminal differentiation of both α- and β-cells. However, β-cells are generated at the onset of *Mafa* expression [14]. *Mafa* knockout animals exhibited overt diabetic symptoms due to malfunctional β-cells, suggesting that *Mafa* is a key transcription factor for β-cells maturation and functions as glucose-responsive cells, while *Mafb* is required for their differentiation [14]. Additional transcription factors necessary for the normal function of mature β-cells, including *Pdx1*, *Pax4*, NK2 homeobox 2 (*Nkx* 2.2), and NK6 homeobox 1 (*Nkx* 6.1), are constitutively expressed [15].

The ability of interconversion between different pancreatic cell types has gained increased research interest as a promising diabetic therapy [16]. Pancreatic injury models have revealed a population of pancreatic progenitors residing near the pancreatic ducts that are positive for NEUROG3 [17]. Newly published research has indicated that these NEUROG3+ cells contribute to β-cell neogenesis, which appears more prominent in the diabetic state [18]. Additionally, fully differentiated acinar cells were switched to β-like cells in response to the forced expression of *Neurog3, Pdx1,* and *Mafa* [19]. Alternatively, in response to severe β-cell loss, α-cells spontaneously shift toward β-cell phenotype [20]. Several drugs, including gamma-aminobutyric acid (GABA) [21] and glucagon-like peptide 1 (GLP-1) [22], have been identified as potential transdifferentiating agents of α- to β-cells.

Natural extracts such as *Zingiber officinale*—commonly known as ginger—is rich in several phytochemicals that have been extensively analyzed, revealing that a-zingiberene, zingerone, gingerols, β-sesquiphellandrene, a-curcumene, a-farnesene, and β-bisabolene are the main constituents of ginger extract [23,24]. The safety and efficiency of ginger extract to ameliorate hyperglycemia have been reported in different studies [25–27]. Aqueous ginger extract treatment significantly alleviated blood glucose level in the streptozotocin (STZ)-induced diabetic rats in a dose- and time-dependent manner, by restoring the glycolytic enzymes activity [28], down-regulating the expression of the pro-inflammatory cytokines, and increasing the total antioxidant capacity [29,30]. Recent systematic reviews are highlighting the ameliorative effects of ginger on diabetes-related complications [31], in which the effect of ginger against the diabetes-associated degenerative changes was matching the effect of metformin [32]. Moreover, the antidiabetic effect of ginger was reported previously by our group in STZ-induced diabetic rats, where ginger-treated diabetic rats displayed ameliorated blood glucose levels, lipid profiles, and kidney function [33]. The reversal of diabetes-related complications is likely to be linked to the regeneration of β-cell mass. To investigate this possibility, we examined the direct effects of aqueous ginger extract treatment on differential pancreatic gene expression in diabetic rats throughout the recovery period. Additionally, to further explore the potential source of insulin-secreting cells, immunofluorescence assessments were performed.

## Results

### Effects of ginger extract on body weight, FBG, and serum insulin levels in STZ-induced diabetic rats

**Figure 1:**
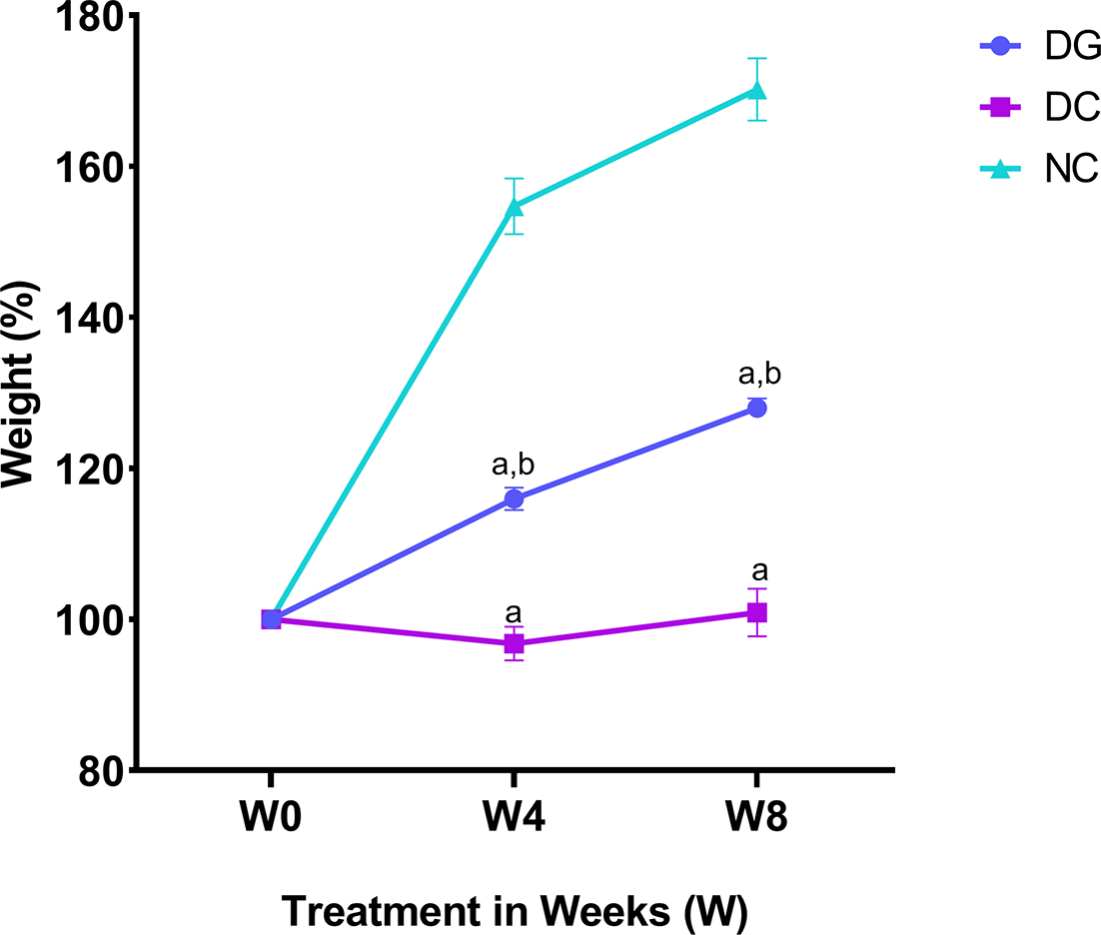
Change in weight percentage across the treatment period in different groups. TheChange in weight percentage across the treatment period in normal control (NC), STZ-induced diabetic control (DC), and ginger extract-treated group (DG). The represented data are the mean values of the weights in each group normalized to 100%, with their standard error of the mean (SEM; *n*=3 rats per group at each time point); **a**:: significant difference compared towith the normal control group, **b**:: significant difference between the diabetic control group given ddH_2_O and diabetic group treated with ginger extract.t.

**Figure 2:**
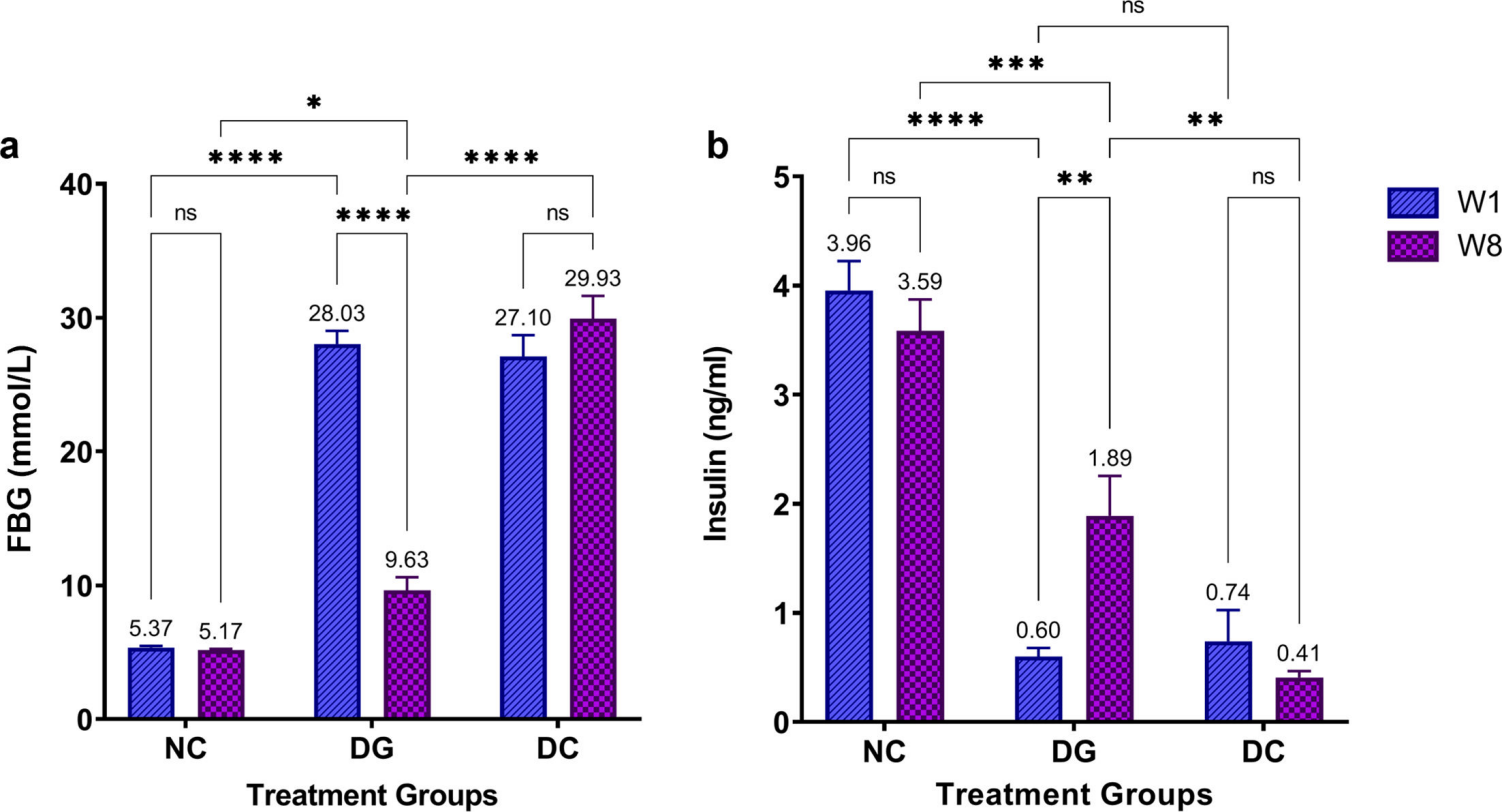
Glucose lowering effect of ginger extract treatment. (**A**) Fasting blood glucose levels (FBG; mmol/LL) and (**B**) serum insulin level (ng/mLl) in normal control (NC), STZ-diabetic control (DC), and ginger extract-treated group (DG) after one and eight weeks. Data represents the mean ± SEM (*n*=3 rats per group at each time point; *P≤0.05, **P≤0.01, ***P≤0.001, **** *P*≤0.0001, ns: *P*>0.05).).

The administration of ginger extract for eight weeks after inducing diabetes resulted in a gradual and significant increase in the body weight of the diabetic-ginger extract-treated group (DG) rats (*P*≤0.0001) compared with that of the diabetic control (DC) rats, while no change in the body weight of DC rats administered double distilled water (ddH_2_O) was observed (Figure 1). After one week of treatment, DG rats displayed highly elevated fasting blood glucose (FBG) levels (27.57 mmol/L) compared with NC rats (5.37 mmol/L; *P*≤0.0001), whereas no significant difference was observed between the DG and DC rats. In contrast, FBG level decreased significantly after eight weeks of ginger extract intake in DG rats (9.633 mmol/L) compared with that in DC rats (29.93 mmol/L; Figure 2a). Concordant with FBG level, serum insulin level significantly increased in DG rats after eight weeks of ginger extract treatment (1.9 ng/ml) compared with that at one week of treatment (0.6 ng/ml; Figure 2b). Moreover, the serum insulin level in the DG group at eight weeks was significantly higher than that in the DC group (0.4 ng/ml; *P*≤0.01).

### Effect of ginger extract treatment on the expression of genes related to islet cells regeneration

**Figure 3:**
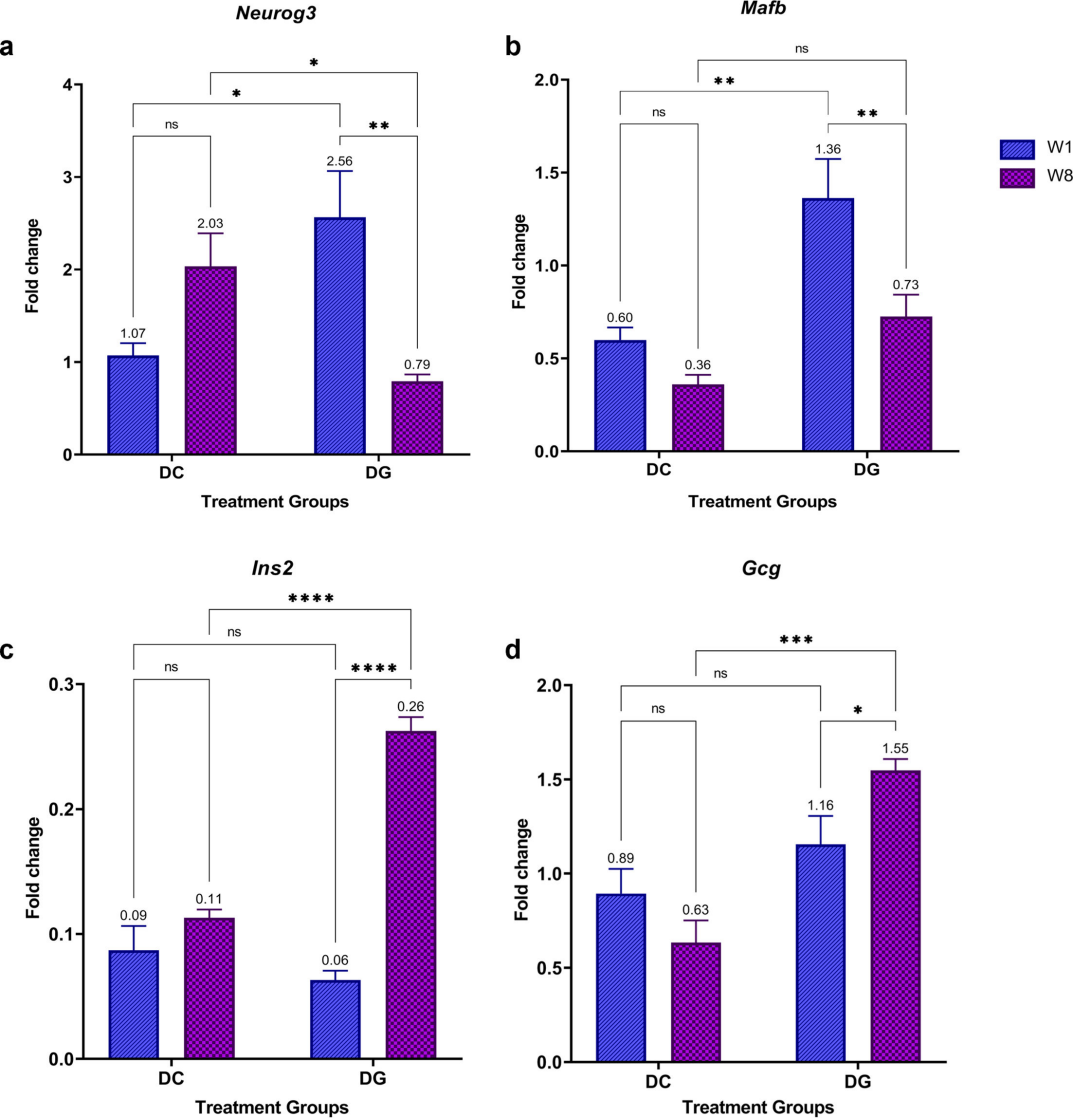
Differential gene expression in STZ-diabetic control (DC) and ginger extract-treated group (DG) after one and eight weeks of treatment. (A**A**) *Neurog3*, (**B**) *Mafb*, (**C**) *Ins2*, and (**D**) *Gcg*, the fold change was determined relative to normal baseline expression. Data represents the mean ± SEM (*n*=3 rats per group at each time point; *P≤0.05, ** *P*≤0.01, ***P≤0.001, ****P≤0.0001, ns: *P*>0.05).).

Ginger intake for one week significantly up-regulated the expression of *Neurog3* (*P*≤0.05) and *Mafb* (*P*≤0.01) in the DG group compared with their expression in the DC group (Figure 3a and b). However, after eight weeks, the expression of *Neurog3* in the DG group significantly declined by 1.24-fold compared with that in the DC group (*P*≤0.05), whereas *Mafb* expression became comparable in both groups (*P*>0.05). Moreover, the expression levels of *Ins2* or *Gcg* in the DC and DG groups did not differ significantly after one week of ginger extract treatment. However, DG rats showed increased expression of both *Ins2* (*P*≤0.0001) and *Gcg* (*P*≤0.001) after eight weeks of treatment with ginger extract (Figure 3c and d).

### Islet size, composition, and the source of insulin-producing cells

**Figure 4:**
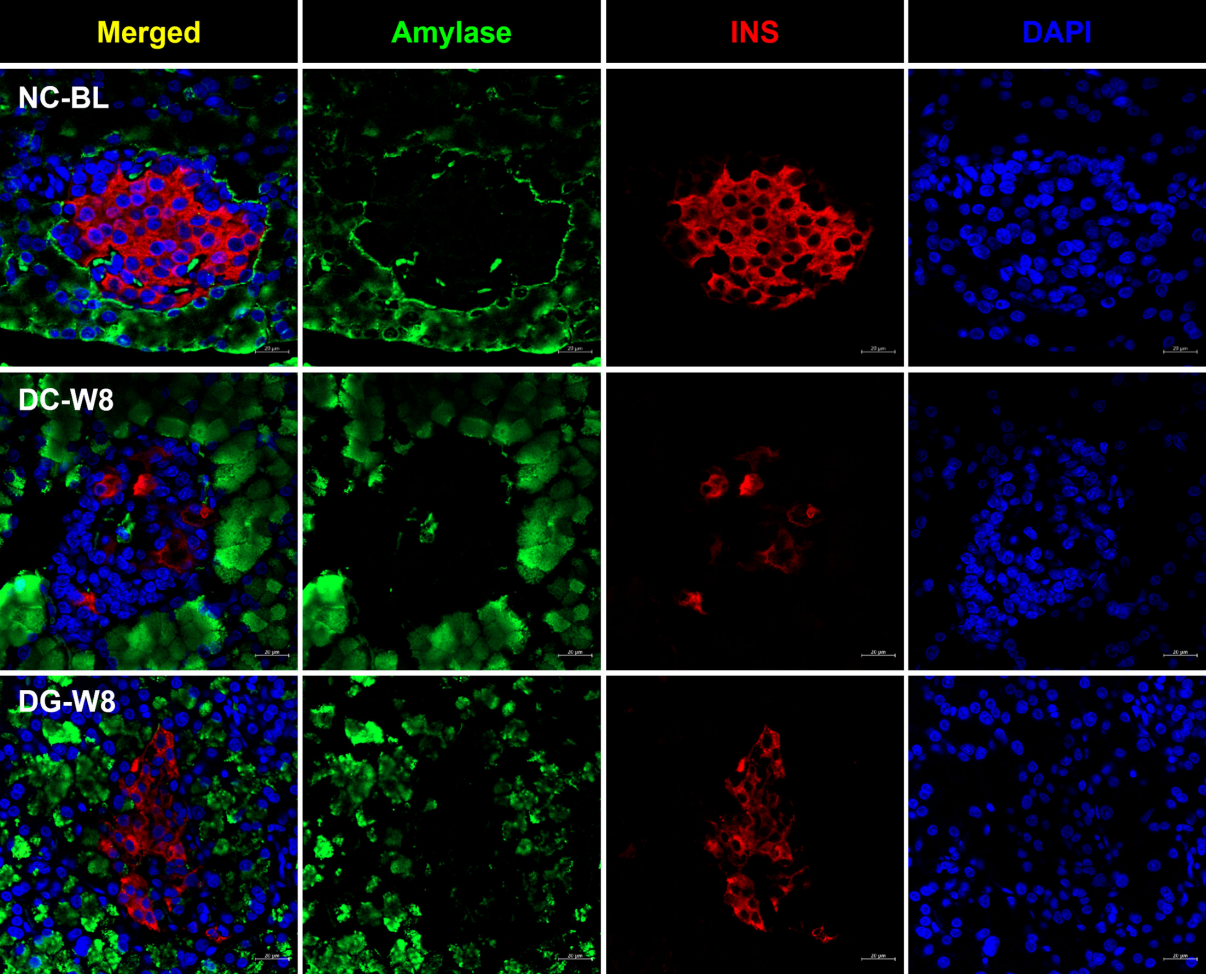
Double immunofluorescent staining of pancreatic tissues for amylase and insulin (INS). DoubleDouble immunofluorescent staining of rat pancreatic tissues for insulin andamylase (green) and INS (red) in baseline normal control rats (NC-BL), diabetic control rats after eight weeks (DC-W8), and diabetic rats treated with ginger extract for eight weeks (DG-W8).).

**Figure 5:**
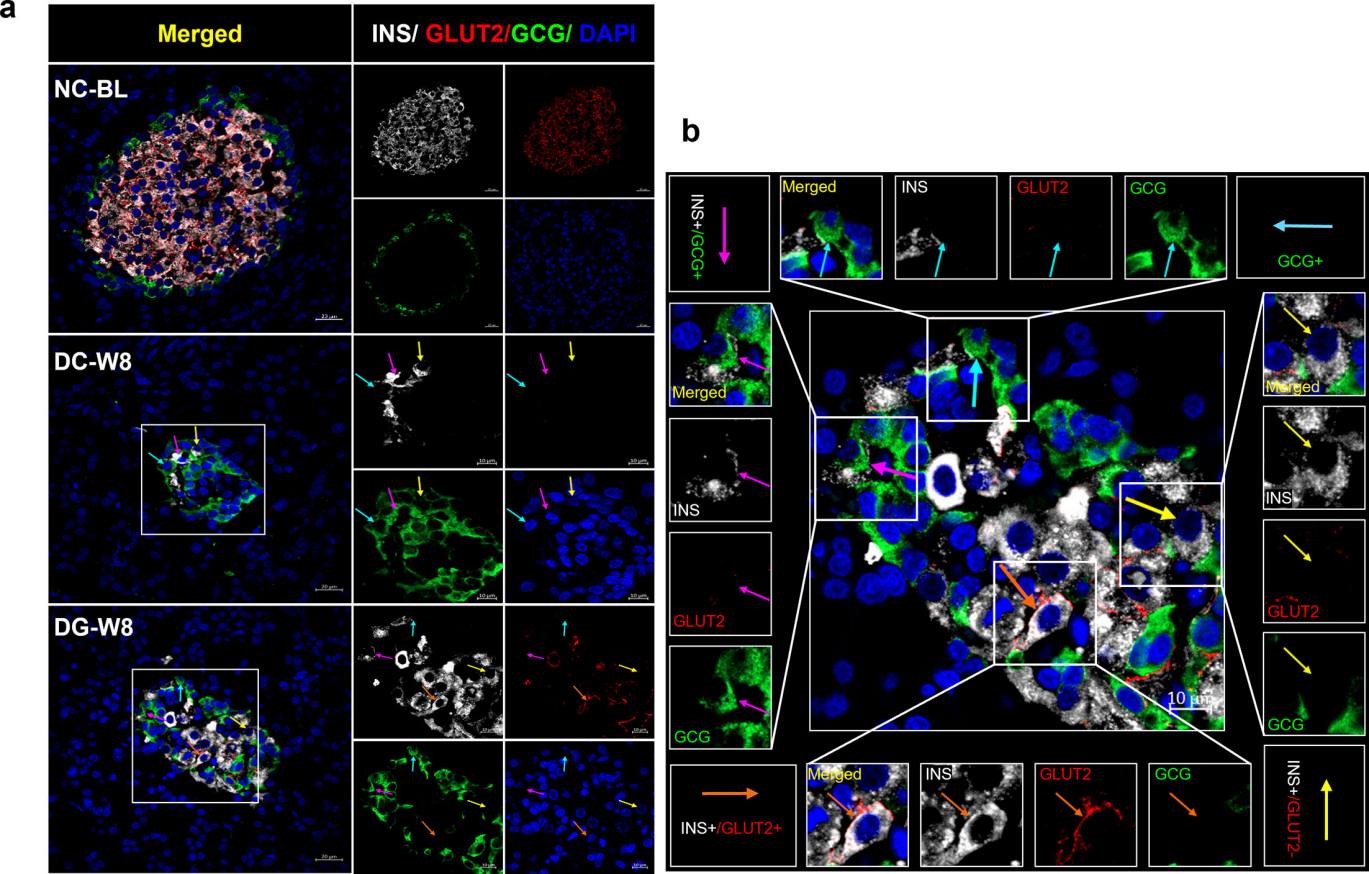
Triple immunofluorescent staining of rat pancreatic tissues for insulin (INS), glucagon (GCG), and glucose transporter 2 (GLUT2). (**A**) Triple immunofluorescent staining of rat pancreatic tissues with insulin (), glucagon (), and glucose transporter 2 (GLUT2) offor INS (white), GCG (green), and GLUT2 (red) in baseline normal control rat (NC-BL), diabetic control rats after eight weeks (DC-W8), and diabetic rats treated with ginger extract for eight weeks (DG-W8). (**B**) Magnified DG-W8 islet showing different islet cell types; pink arrow: INS+/GCG+ cell; orange arrow: INS+/GLUT2+ mature β-cell; blue arrow: GCG+ α-cell; yellow arrow: INS+/GLUT2-− immature β-cells.

The fixed pancreata were initially co-stained with amylase and insulin to identify the origin of the newly formed insulin-producing cells. We investigated the pancreatic cell source of insulin in the DG group and explored the possibility of acinar to β-cell conversion. No colocalization was observed between the two markers in any of the groups, as INS expression was only observed in the islets, and amylase was restricted to the surrounding acinar cells (Figure 4). Furthermore, DC rats displayed severe morphological disturbances in the pancreatic islets compared with NC rats. To further investigate the islet cell insulin source, triple staining was performed for INS, GCG (as an α-cell marker), and glucose transporter 2 (GLUT2) expressed only in mature β-cells. The pancreas of the DC group had very few small islets after eight weeks, with an abnormal islet cell distribution compared with that of the NC group. GCG+ α-cells of the DC rats were grouped in the center of the islets instead of being organized at the periphery with some cells co-expressing GCG and INS (Figure 5a). Conversely, in the islets of the DG group, mature β-cells co-expressing INS and GLUT2 and GCG+ α-cells were identified. A few cells expressed only INS or both GCG and INS (Figure 5b).

**Figure 6:**
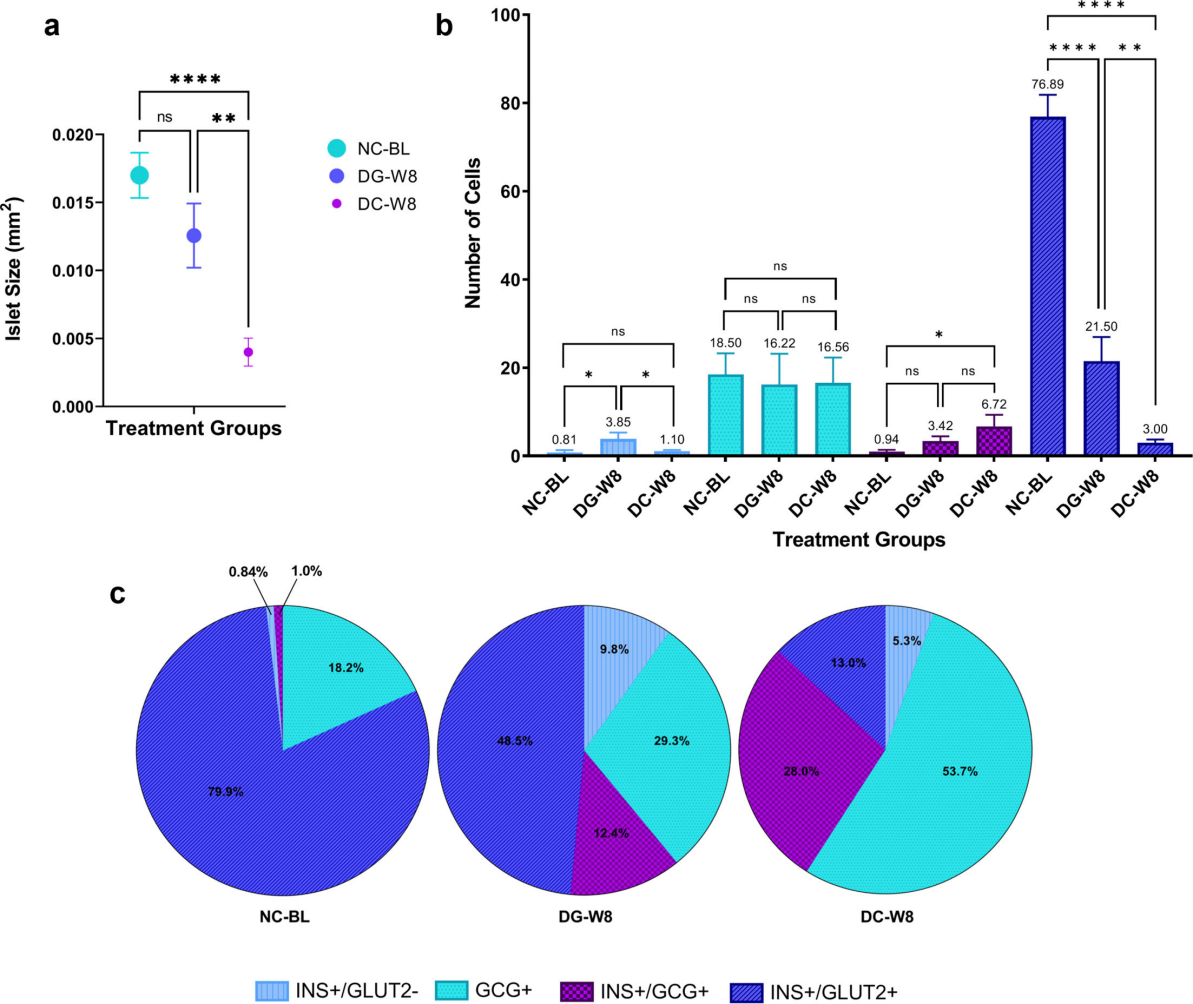
Pancreatic islets morphology and cellular composition in different groups. (**A**) the iIslet size (mm^2^; *n*=2 rats per group; 7 islet images were collected from each rat), (**B**) the number of different cell types (*n*=2 rats per group; 3 sections per rat), and (**C**) the percentage of each counted cell type within the islet of baseline normal control rats (NC-BL), and in STZ-induced diabetic rats after eight weeks of ddH_2_O (DC) or ginger extract treatment (DG) (*n*=2 rats per group; 3 sections per rat; *P≤0.05, ** *P*≤0.01, ***P≤0.001, and ****P≤0.0001).

To better understand the effects of ginger extract treatment on pancreatic islet size and composition, we compared the variations in islet size and cellular composition in each group by counting four different cell types (GCG+, INS+/GCG+, INS+/GLUT2−, and INS+/GLUT2+; Figure 6). The results revealed significantly smaller sized islets in DC rats than in NC-BL rats (Figure 6a), with a significant reduction in the number of mature β-cells in the islets of DC rats compared with that in the NC-BL rats (*P*≤0.001), making only 13.0% of the counted cells. In contrast, the number of α-cells was comparable (*P*≥0.05) between the NC and DC groups (Figure 6b); however, α-cells made up more than 50% of the counted cells in islets of DC rats (Figure 6c). Additionally, the DC group displayed the largest number of INS+/GCG+ cells, which was significantly higher than that in NC-BL group (*P*≤0.05) and comprised 28% of the counted islet cells. In contrast, the DG group displayed significantly (*P*≤0.01) larger islets in comparison with DC rats (Figure 6a). Furthermore, the islets of DG rats exhibited a significant rise in the number of mature INS+/GLUT2+ β-cell population compared with the DC group comprising the highest percentage (48.5%) of the counted islet cells (Figure 6b and c). Additionally, the number of immature INS+/GLUT2− β-cells was significantly elevated in the DG group, comprising 9.8% of the counted cells compared with the DC group (Figure 6b and c). In contrast, the numbers of GCG+ α-cells and INS+/GCG+ cells did not differ between the DC and DG groups. Nevertheless, the islet percentages of GCG+ and INS+/GCG+ cells were reduced by 24.4% and 15.6%, respectively, in the DG group compared with those in the DC group (Figure 6c).

## Discussion

The efficacy of ginger as an antidiabetic agent has been extensively established both in diabetic animal models and in human patients [34–36], suggesting the potent protective and regenerative properties of ginger on pancreatic islets [37]. Therefore, evaluating the direct effects of ginger on pancreatic islet architecture and gene expression would expand our understanding of the mechanisms underlying its antidiabetic effects. Herein, we report that ginger extract ameliorated diabetes, which was reflected by preventing weight loss, lowering glycemia, and increasing serum insulin levels, possibly through the stimulation of β-cell neogenesis.

The direct effect of ginger extract on the differential expression of key transcription factors involved in islet cell differentiation in diabetic animals revealed that one week of ginger treatment was sufficient to induce a significant rise in the expression of *Neurog3* and *Mafb*. Continuing treatment for eight weeks significantly diminished their mRNA levels, mirroring the natural developmental process of normal murine pancreatic islets. *Neurog3* is a pro-endocrine gene indispensable for the generation of all endocrine lineages, including β-cells [10]. During pancreatic islet differentiation, *Neurog3* mRNA expression peaks at E15.5, indicating the formation of endocrine progenitors, and its expression is reduced postnatally in the adult pancreas [38]. Consistent with the previous studies, the reduction in *Neurog3* expression observed in the diabetic recovery model in the present study indicates that ginger extract treatment could induce putative pancreatic islet endocrine progenitors to eventually give rise to insulin-producing cells. A similar expression pattern of *Neurog3* was reported in the garlic-induced recovery of STZ-diabetic rats [39,40] and in other studies where the up-regulation of *Neurog3* was observed during β-cell mass regeneration [41,42]. In contrast, *Mafb* is expressed later in both α- and β-cell progenitors [13]. In β-cells, early expression of *Mafb* induces the expression of several key genes required for glucose homeostasis, including *Ins1, Ins2*, *Slc2a2* (GLUT2 encoding gene), and *Mafa* which replaces *Mafb* in adult β-cells to maintain the expression of these genes [11,43]. Recently, Bsharat et al. [44] revealed an additional role of *Mafb* in promoting proper islet morphogenesis and β-cell clustering. Together with these findings, decreased *Mafb* expression after eight weeks of ginger extract treatment in this study could be because of increased *Mafa* expression, leading to pancreatic islet regeneration and β-cell neogenesis.

The significantly altered expression of *Ins2* and *Gcg* was observed only after eight weeks of ginger extract treatment. The up-regulation of *Ins2* in the DG group compared with the DC counterparts was consistent with the significant increase in serum insulin levels and reduced FBG. *Gcg* encoding proglucagon was also up-regulated significantly in the DG group. In pancreatic α-cells, proglucagon is mainly processed into GCG, which increases blood glucose levels during hypoglycemia. However, α-cells also play several roles in the regulation of blood glucose levels [45]. Recent evidence suggests a paradoxical effect of GCG on maintaining normoglycemia, suggesting an insulinotropic effect of GCG [46]. However, the stimulatory effect of GCG on insulin release occurs only at high glucose levels; therefore, it potentiates glucose-stimulated insulin secretion (GSIS) rather than initiating insulin secretion [47]. Moreover, in the developing pancreas and under certain conditions, such as pregnancy and hyperglycemia, pancreatic α-cells can induce GLP-1 production, which induces insulin production and β-cell regeneration [48,49]. These findings suggest that α-cells play an essential role in the recovery of the diabetic model. Taken together, we suggest that the ginger extract-mediated increase in *Gcg* expression might boost the intra-islet content of GCG or its related peptides from α-cells to exert beneficial effects on neighboring β-cells by restoring their mass and modulating GSIS.

The plasticity of pancreatic cells to compensate for β-cell loss has been previously demonstrated [7,50]. Therefore, we explored the potential of both exocrine and endocrine cells to give rise to β-cells in response to ginger intake since both cell types originate from the same progenitor cell pool during early embryogenesis [51]. As exocrine cells are the most abundant pancreatic cell type and were reported to give rise to insulin-producing cells in diabetic animal models, they were considered a potential source for β-cell neogenesis [52]. However, in the present study, ginger extract treatment was insufficient to induce cell interconversion, as amylase-expressing cells did not show any colocalization of amylase and insulin.

Furthermore, as both α- and β-cells develop from NEUROG3+ endocrine progenitors, we examined the possibility of α- to β-cell transdifferentiation, as α-cells seem to be more attractive source of neo-β-cells and have always been viewed as the ‘close relatives’ to β-cells [53]. Immunofluorescence staining of the pancreatic tissue revealed that DC rats displayed significantly smaller pancreatic islets due to the ablation of most mature β-cell population [54], causing GCG+ α-cells to dominate and make up more than 50% of the islets. In the same control diabetic model, another group of α-cells was also found to express both GCG and INS. These cells were the second-most dominant in the DC group after α-cells, comprising almost 28% of the counted pancreatic islet cells indicating that STZ-induced pancreatic injury and lack of β-cell population could trigger the spontaneous conversion of α- to β-cells [20]. However, treatment with ginger extracts significantly restored islet size, as reported previously [37]. This could be mainly due to the significant increase in mature β-cell number, as seen by the emergence of a cell population in the center of the islet co-expressing two mature cell markers, INS+/GLUT2+. These cells were the most abundant, comprising 48.5% of the counted islet cell types, shifting the islet cell distribution toward a more normal morphology. The newly generated mature β-cells may have originated from α-cells through an intermediate stage of INS+/GCG+ double positive cells, followed by INS+/GLUT2− immature β-cells, which were found mostly in the DG group. These immature β-cells, in turn, may have progressed toward mature INS+/GLUT2+ β-cells as ginger extract treatment continued for eight weeks, suggesting a second role of α-cells in maintaining proper islet functioning as reported in several studies utilizing antidiabetic drugs [21,50,55]. In fact, the up-regulation of *Neurog3* and *Mafb* after the first week may also have activated the formation of α-cell from pancreatic progenitors to compensate for the transdifferentiated population [20], which could explain the comparable numbers of α-cells in all groups, regardless of the transdifferentiation process. While the results do suggest that the newly generated β-cells may have been generated from the pancreatic endocrine progenitors and/or α- to β-cell transdifferentiation, verifying the results with a larger sample size and exploring the effect of ginger extract on glycated hemoglobin, antioxidant status, and inflammatory markers, in addition to the islet content of GCG and GLP-1, would provide more in-depth insight into the exact mechanism underlying the antidiabetic effect of ginger extract.

**Figure 7:**
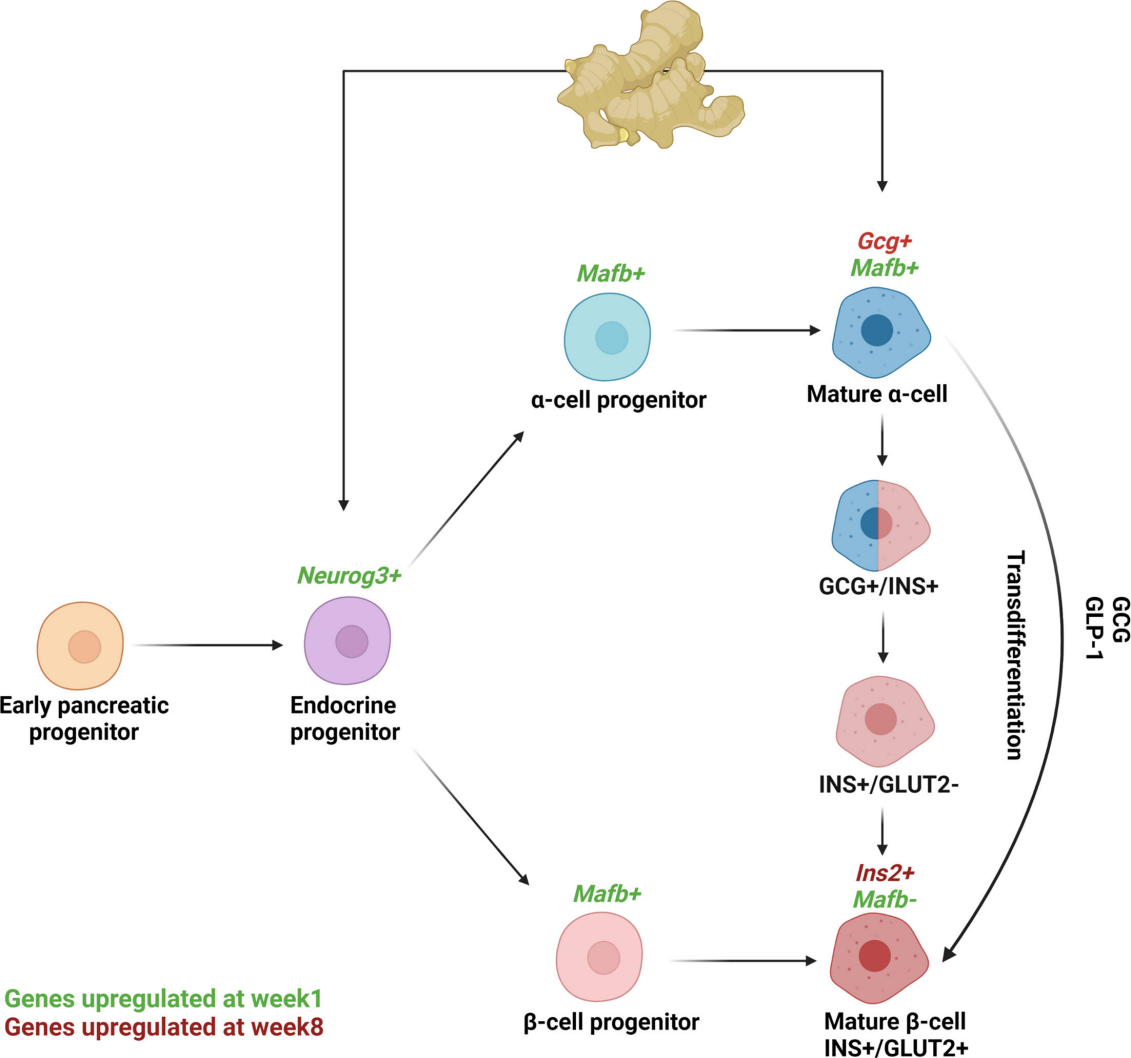
Proposed antidiabetic mechanism of ginger extract acting on pancreatic endocrine progenitors and α-cells. Created with BioRender.com, accessed on 12 September 2023.

In conclusion, ginger extract efficiently induced the recovery of diabetic animals through pancreatic islet reconstruction, leading to increased serum insulin levels through the neogenesis of insulin- and glucagon-producing cells. The findings suggest that ginger extract induces recapitulation of the pancreatic endocrine developmental process one week after its administration by up-regulating the expression of *Neurog3,* thereby inducing the formation of endocrine progenitors that eventually give rise to all endocrine lineages. Additionally, it up-regulated *Mafb*, expressed during endocrinogenesis in both α- and β-cell progenitors, eventually giving rise to newly differentiated β-cells and probably α-cells. A population of α-cells may participate in replenishing β-cells pool via direct transdifferentiation. In addition, ginger could influence α-cell to play an additional role in restoring β-cell function by up-regulating *Gcg*. Collectively, the islets of the ginger-treated rats had an almost similar size and architecture to their normal counterparts. Furthermore, the newly generated β-cells produced and secreted insulin as seen by the rise in its mRNA expression and serum levels. Collectively, we demonstrate that ginger extract acts on pancreatic progenitors and α-cells to restore β-cell mass in STZ-induced diabetic rats (Figure 7).

This study uncovered the potential use of ginger as a natural, safe, and unexpensive antidiabetic drug to trigger the endogenous β-cell regeneration in the treated diabetic animals. Understanding the underlying mechanism, the specific genes and pathways involved in inducing the recovery process would help in advancing the current therapeutic strategies to overcome diabetes. Additionally, revealing how new beta cells can be re-created would improve the quality of the patients’ life and minimize the reliance on exogenous insulin. Although the use of STZ-induced animal model of diabetes is one of the most used methods to identify possible antidiabetic agents, it has some drawbacks including the untargeted toxicity to other organs [56], and the lack of other characteristics of human T1DM such as the pancreatic insulitis, especially when a single high dose of STZ is given [57]. Therefore, discrepancies between human and animal models of diabetes should be taken into consideration.

## Methods

### Preparation of aqueous ginger extract treatment

Fresh Indian ginger rhizome (*Zingiber officinale*) was purchased from the local supermarket and used to prepare an aqueous ginger extract as reported previously [33]. Briefly, 150 g of fresh ginger was peeled and homogenized in 50 mL of cold ddH_2_O. The homogenate was filtered and then centrifuged at 15,000 rpm for 15 min, and the aqueous layer was completed to 300 ml with ddH2O. Ginger extract treatment was administered daily as a single oral dose (500 mg/kg body weight).

### Animal model and treatment

Adult male Sprague–Dawley rats (*n*=27, weighing 150–190 g; parents were acquired from B and K Universal Co., Hull, U.K.) were used in this study. The rats were housed in the Animal Breeding and Care Unit, Department of Biological Science, Kuwait University. Animal handling protocols and procedures were performed in accordance with the Instructional Guide for the Care and Use of Laboratory Animals [58] and were approved by the Department of Biological Sciences Ethical Committee for the Use of Laboratory Animals (DBS/IRB(ECULA)23–009). The rats were intraperitoneally injected with STZ (Sigma-Aldrich, St. Louis, MO, U.S.A., cat: S0130-1G) at a dose of 60 mg/kg body weight dissolved in citrate buffer (0.01 M, pH 4.5) after 2 h of fasting to establish a type 1 diabetic model [33]. Overnight FBG levels were measured by tail puncture one week after inducing diabetes using an Accu-Chek Performa glucometer (Roche Diagnostics GmbH, Mannheim, Germany). Rats with FBG levels >12 mmol/L were considered diabetic. The FBG and body weight of all rats were measured throughout the experimental period. The rats were divided into three groups as follows: normal control rats administered ddH_2_O (NC; *n*=11), diabetic control rats administered ddH_2_O (DC; *n*=8), and diabetic-ginger extract-treated rats (DG; *n*=8). The rats in all groups were treated for 8 weeks.

### Rats’ euthanasia and tissue sample collection

Three NC rats were sacrificed at the beginning of the experiment (day 0) for measurement of baseline (BL) characteristics, while three rats from each group (NC, DC, and DG) were sacrificed after the first and eighth weeks of the experiment. The rats were anesthetized using 9:1 Ketamine (10%, Dutch farm Nedar, Host den Berg, Holland): Xylazine (10%, Interchemi.e. Vernary, Holland) mix at a dose of (0.2 ml/100 g of body weight) intraperitonially. Once all indications of sedation were confirmed, the rats were killed by a cardiac puncture to collect blood samples. The pancreatic samples were collected for total RNA extraction following a previously described method [59]. The bile duct was clamped where it enters the duodenum at the sphincter of Oodi. Furthermore, the other end of the bile duct was hold and clamped by a forceps to prevent the retrograde perfusion, and 2 mL of RNA*later*® (Qiagen, Hilden, Germany, cat: 76106) was injected into the bile duct following cannulation near the liver toward the pancreatic duct. Following perfusion, approximately 50–70 mg of the pancreas was excised and placed in 5 mL of cold QIAzol (QIAGEN, Hilden, Germany, cat: 79306) for immediate homogenization using a TissueRuptor Ultra-Turrax T8 (IKA Laboratories, Staufen, Germany).

### Determination of serum insulin concentration

Blood samples were allowed to clot and centrifuged at 3000×g for 10 min to isolate the serum. Serum insulin levels were measured using a rat insulin enzyme-linked immunosorbent assay (ELISA) kit (SPI Bio, Bertin-Pharma, Montigny Le Bretonneux, France, cat: 589501). The assay was performed in duplicate, following the manufacturer’s instructions, and the plate was read at 410 nm using a ClARIOstar microplate reader (BMG Labtech, Ortenberg, Germany).

### Pancreatic RNA extraction

Total pancreatic RNA was extracted directly from the pancreatic homogenate using Qiagen RNeasy Plus Universal Mini Kit following the manufacturer’s instructions (QIAGEN, Hilden, Germany, cat: 73404). The RNA samples were stored directly at −80°C for further analysis. The concentration and purity ratio (A260/A280) of RNA were determined using a NanoDrop-8000 spectrophotometer (Thermo Fisher Scientific, Waltham, MA, U.S.A.). The integrity of the extracted RNA was measured using the Agilent RNA 6000 Nano Kit (Agilent Technologies, Santa Clara, CA, U.S.A., cat: 50671511).

### RNA to cDNA conversion

RNA samples (*n*=15; 3 from NC-BL, 3 each from DG and DC at weeks 1 and 8) were diluted to a concentration of 280 ng/ml. Furthermore, RNA samples were reverse-transcribed into cDNA using SuperScript^®^ IV First-Strand Synthesis System (Invitrogen, Carlsbad, CA, U.S.A., cat: 18091050) following the manufacturer’s instructions. The RNA–primer mix was prepared by mixing 50 μM oligo d(T)_20_ primer and 10 mM dNTPs (1 μl each), DEPC-treated water (4 μl), and sample RNA (7 μl). The mix was heated at 65°C for 5 min and incubated on ice for 1 min. The reverse transcription reaction mix was prepared by adding 4 μl 5X SSIV buffer, 1 μl each of 100 mM DTT RNase inhibitor and SSIV reverse transcriptase. The reaction mix was added to the annealed RNA and incubated initially at 23°C for 10 min and then at 50–55°C for 10 min. Finally, the mixture was incubated at 80°C for 10 min. The concentration of cDNA was measured using NanoDrop-8000 (Thermo Fisher Scientific) and diluted to a final concentration of 50 ng/µL for quantitative real-time PCR (qRT-PCR).

### qRT-PCR

The expression of four genes including insulin 2 (*Ins2),* glucagon (*Gcg), Mafb,* and *Neurog3,* in addition to actin-beta (*Actb*) as an endogenous control, was assessed using the TaqMan Gene Expression Assay kit (Applied Biosystems, Foster City, CA, U.S.A., cat: 4369016) following the manufacturer’s instructions. The probes used for the target gene were labeled with fluorescein amides (FAM, blue), and those for *Actb* were labeled with Aequorea Victoria (VIC, green) fluorescent reporter dyes for duplex PCR assays. The identification number of the assays is listed in Table 1. The master mix was prepared by adding 1 μl of each primer for the target gene and *Actb*, 10 μl PCR mix, 5 μl nuclease-free water, and 3 μl of each sample. The master mix was added to the corresponding sample well in a 96-well plate. Plate readings were performed using the ViiA 7 Real-Time PCR system and QuantStudio Real-Time PCR software (Applied Biosystems, Foster City, CA, U.S.A.). The assays were performed in duplicate, and the thermal conditions were set as follows: 50°C for 2 min, 95°C for 10 min, followed by 40 cycles of 95°C for 15 sec, and 60°C for 1 min.

**Table 1:** Primers for qRT-PCR.

Target gene	Assay ID number
*Ins2*	Rn02121433_g1
*Gcg*	Rn00562293_m1
*Mafb*	Rn00709456_s1
*Neurog3*	Rn00572583_s1
*Actb*	Rn00667869_m1

### Immunohistochemistry (IHC)

Pancreatic tissues from NC-BL, DG, and DC rats were collected after eight weeks of treatment, fixed with 4% paraformaldehyde (Merck, Darmstadt, Germany, cat: 104005) and embedded in paraffin wax. The blocks were then sliced into 4-μm-thick sections, deparaffinized, and rehydrated in a descending series of ethanol. To permeabilize the tissues, sections were incubated with 1% Triton X-100 (Sigma-Aldrich, St. Louis, MO, U.S.A.) for 30 min. Antigen retrieval was performed using a Tris-EDTA solution (pH 9) at 90°C (Tris, BioRad, Richmond, CA, U.S.A., cat: 1610719; EDTA , Alpha Chemika, Mumbai, India, cat: AL1641). The sections were blocked using 2% blocking buffer (Roche, Mannheim, Germany, cat: 11096176001) for 30 min and then incubated overnight at 4°C with the following diluted primary antibodies: guinea pig anti-insulin polyclonal antibody (Progen Biotechnik, cat: 16049, 1∶400), rabbit anti-insulin antibody (Abcam, cat: Ab181547, 1:400), mouse monoclonal anti-glucagon antibody (Abcam, cat: ab10988, 1:150), rabbit anti-GLUT2 polyclonal antibody (Sigma Aldrich, cat: 07–1402, 1:150), and mouse monoclonal anti-amylase antibody (Santa Cruz Biotechnology, cat: SC-46637(G-10), 1:200). Slides were washed in phosphate-buffered saline (PBS) (Sigma-Aldrich, St. Louis, MO, U.S.A., cat: P4417) and incubated for 30 min with the fluorophore-conjugated secondary antibody produced in goats anti-mouse FITC green (Sigma, cat: F0257-1ML, 1:100), anti-guinea pig Alexa Fluor 647 (Invitrogen, cat: A-21450, 1:400), and anti-rabbit Alexa Fluor 594 (Invitrogen, cat: A-32740, 1:400). The slides were washed again in PBS, mounted with DAPI containing mounting media (Abcam, Cambridge, England, cat: ab104139), and viewed under a ZEISS LSM 800 META confocal laser-scanning microscope (Carl Zeiss Imaging, Germany). Images were captured using Zen 2.3 software (Zeiss, Oberkom, Germany). Different cell types from three sections of each rat (*n*=2 for each group) were counted manually using ImageJ software, with three islets per section. Islet size was determined based on seven islet images (three to four islets from each rat) per group.

### Statistical analysis

Two-way analysis of variance (ANOVA) was performed using GraphPad Prism 9.3.1 (GraphPad Software, Inc., San Diego, CA, U.S.A.) to analyze FBG, body weight differences, serum insulin levels, and qRT-PCR results for all animal groups. qRT-PCR data were analyzed using the comparative CT method (2**^- Δ Δ Ct^**; Ct: the threshold cycle) to represent the fold change in gene expression levels in relation to normal baseline control. One-way ANOVA was used to analyze the differences in islet size and cell number. The normal distribution of the samples was tested using Shapiro–Wilk and Kolmogorov–Smirnov tests. Statistical significance was set at *P*≤0.05

## Data Availability

The datasets used and/or analyzed during the current study are available from the corresponding author upon reasonable request.

## Abbreviations

ANOVA: Analysis of variance
BL: baseline
DC: Diabetic control
DC: diabetic control
DG: Diabetic treated with ginger
ELISA: enzyme-linked immunosorbent assay
FBG: fasting blood glucose
GABA: gamma-aminobutyric acid
GLP-1: glucagon like peptide 1
GLUT2: Glucose transporter 2
GSIS: glucose-stimulated insulin secretion
Gcg: Glucagon
IHC: Immunohistochemistry
Ins: Insulin
MAF: musculoaponeurotic fibrosarcoma
NC: Normal control
Neurog3: Neurogenin-3
Pdx1: pancreatic and duodenal homeobox 1
SEM: standard error of the mean
STZ: streptozotocin
Slc2a2: solute carrier family 2 member 2
T1DM: Type 1 Diabetes Mellitus
ddH_2_O: double distilled Water

## References

[1] Saeedi P., Petersohn I., Salpea P., Malanda B., Karuranga S., Unwin N. (2019). Global and regional diabetes prevalence estimates for 2019 and projections for 2030 and 2045: results from the international diabetes federation diabetes atlas, 9th edition. Diabetes Res. Clin. Pract..

[2] Dludla P.V., Mabhida S.E., Ziqubu K., Nkambule B.B., Mazibuko-Mbeje S.E., Hanser S. (2023). Pancreatic β-cell dysfunction in type 2 diabetes: implications of inflammation and oxidative stress. World J. Diabetes.

[3] Michalek D.A., Onengut-Gumuscu S., Repaske D.R., Rich S.S (2023). Precision medicine in type 1 diabetes. J. Indian Inst. Sci..

[4] Vettoretti M., Facchinetti A (2019). Combining continuous glucose monitoring and insulin pumps to automatically tune the basal insulin infusion in diabetes therapy: a review. Biomed. Eng. Online.

[5] Niu F., Liu W., Ren Y., Tian Y., Shi W., Li M. (2024). β-cell neogenesis: a rising star to rescue diabetes mellitus. J. Adv. Res..

[6] Mastracci T.L., Sussel L (2012). The endocrine pancreas: insights into development, differentiation, and diabetes. Wiley Interdiscip. Rev. Dev. Biol..

[7] Zhu Y., Liu Q., Zhou Z., Ikeda Y (2017). PDX1, neurogenin-3, and MAFA: critical transcription regulators for beta cell development and regeneration. Stem Cell Res. Ther..

[8] Smith S.B., Gasa R., Watada H., Wang J., Griffen S.C., German M.S (2003). Neurogenin3 and hepatic nuclear factor 1 cooperate in activating pancreatic expression of Pax4. J. Biol. Chem..

[9] Grapin-Botton A., Majithia A.R., Melton D.A (2001). Key events of pancreas formation are triggered in gut endoderm by ectopic expression of pancreatic regulatory genes. Genes Dev..

[10] Artner I., Le Lay J., Hang Y., Elghazi L., Schisler J.C., Henderson E. (2006). MafB: an activator of the glucagon gene expressed in developing islet alpha- and beta-cells. Diabetes.

[11] Hang Y., Stein R (2011). MafA and MafB activity in pancreatic β cells. Trends Endocrinol. Metab..

[12] Nishimura W., Kondo T., Salameh T., El Khattabi I., Dodge R., Bonner-Weir S. (2006). A switch from MafB to MafA expression accompanies differentiation to pancreatic beta-cells. Dev. Biol. (NY).

[13] Xiafukaiti G., Maimaiti S., Ogata K., Kuno A., Kudo T., Shawki H.H. (2019). MafB is important for pancreatic β-cell maintenance under a MafA-deficient condition. Mol. Cell. Biol..

[14] Conrad E., Dai C., Spaeth J., Guo M., Cyphert H.A., Scoville D. (2016). The MAFB transcription factor impacts islet α-cell function in rodents and represents a unique signature of primate islet β-cells. Am. J. Physiol. Endocrinol. Metab..

[15] Saleh M., Gittes G.K., Prasadan K (2021). Alpha-to-beta cell trans-differentiation for treatment of diabetes. Biochem. Soc. Trans..

[16] Wang K.L., Tao M., Wei T.J., Wei R (2021). Pancreatic β cell regeneration induced by clinical and preclinical agents. World J. Stem Cells.

[17] Xu X., D’Hoker J., Stangé G., Bonné S., De Leu N., Xiao X. (2008). Beta cells can be generated from endogenous progenitors in injured adult mouse pancreas. Cell.

[18] Gribben C., Lambert C., Messal H.A., Hubber E.L., Rackham C., Evans I. (2021). Ductal Ngn3-expressing progenitors contribute to adult β cell neogenesis in the pancreas. Cell Stem Cell.

[19] Clayton H.W., Osipovich A.B., Stancill J.S., Schneider J.D., Vianna P.G., Shanks C.M. (2016). Pancreatic inflammation redirects acinar to β cell reprogramming. Cell Rep..

[20] Thorel F., Népote V., Avril I., Kohno K., Desgraz R., Chera S. (2010). Conversion of adult pancreatic alpha-cells to beta-cells after extreme beta-cell loss. Nature.

[21] Ben-Othman N., Vieira A., Courtney M., Record F., Gjernes E., Avolio F. (2017). Long-term GABA administration induces alpha cell-mediated beta-like cell neogenesis. Cell.

[22] Lee Y.S., Lee C., Choung J.S., Jung H.S., Jun H.S (2018). Glucagon-like peptide 1 increases β-cell regeneration by promoting α- to β-cell transdifferentiation. Diabetes.

[23] El-Bahr S.M., Elzoghby R.R., Alfattah M.A., Kandeel M., Hamouda A.F (2022). Aqueous ginger (*Zingiber officinale*) extract ameliorates the harmful effects of high-dose lornoxicam in albino male rats. Biomed Res. Int..

[24] Spyrou A., Batista M.G.F., Corazza M.L., Papadaki M., Antonopoulou M (2024). Extraction of high value products from *Zingiber officinale* roscoe (Ginger) and utilization of residual biomass. Molecules.

[25] Mao Q.Q., Xu X.Y., Cao S.Y., Gan R.Y., Corke H., Beta T. (2019). Bioactive compounds and bioactivities of ginger (*Zingiber officinale* Roscoe). Foods.

[26] Rong X., Peng G., Suzuki T., Yang Q., Yamahara J., Li Y (2009). A 35-day gavage safety assessment of ginger in rats. Regul. Toxicol. Pharmacol..

[27] Faddladdeen K.A.J (2022). The possible protective and therapeutic effects of ginger and cinnamon on the testis and coda epididymis of streptozotocin-induced-diabetic rats: histological and biochemical studies. Saudi J. Biol. Sci..

[28] Abdulrazaq N.B., Cho M.M., Win N.N., Zaman R., Rahman M.T (2012). Beneficial effects of ginger (*Zingiber officinale*) on carbohydrate metabolism in streptozotocin-induced diabetic rats. Br. J. Nutr..

[29] Al-Shathly M.R., Ali S.S., Ayuob N.N (2020). *Zingiber officinale* preserves testicular structure and the expression of androgen receptors and proliferating cell nuclear antigen in diabetic rats. Andrologia.

[30] ALmohaimeed H.M., Mohammedsaleh Z.M., Batawi A.H., Balgoon M.J., Ramadan O.I., Baz H.A. (2021). Synergistic anti-inflammatory and neuroprotective effects of *Cinnamomum cassia* and *Zingiber officinale* alleviate diabetes-induced hippocampal changes in male albino rats: structural and molecular evidence. Front. Cell Dev. Biol..

[31] Veisi P., Zarezade M., Rostamkhani H., Ghoreishi Z (2022). Renoprotective effects of the ginger (*Zingiber officinale*) on diabetic kidney disease, current knowledge and future direction: a systematic review of animal studies. BMC Complement. Med. Ther..

[32] Alshathly M.R (2019). Efficacy of ginger (*Zingiber officinale*) in ameliorating streptozotocin-induced diabetic liver injury in rats: histological and biochemical studies. J. Microsc. Ultrastruct..

[33] Al-Amin Z.M., Thomson M., Al-Qattan K.K., Peltonen-Shalaby R., Ali M (2006). Anti-diabetic and hypolipidaemic properties of ginger (*Zingiber officinale*) in streptozotocin-induced diabetic rats. Br. J. Nutr..

[34] Ma H., Li J (2022). The ginger extract could improve diabetic retinopathy by inhibiting the expression of e/iNOS and G6PDH, apoptosis, inflammation, and angiogenesis. J. Food Biochem..

[35] Rostamkhani H., Veisi P., Niknafs B., Jafarabadi M.A., Ghoreishi Z (2023). The effect of *Zingiber officinale* on prooxidant-antioxidant balance and glycemic control in diabetic patients with ESRD undergoing hemodialysis: a double-blind randomized control trial. BMC Complement. Med. Ther..

[36] Sharma S., Shukla M.K., Sharma K.C., Tirath K., Kumar L., Anal J.M.H. (2023). Revisiting the therapeutic potential of gingerols against different pharmacological activities. Naunyn Schmiedebergs Arch. Pharmacol..

[37] Mohamed N., Abd El-Samei M., Abd El-ghaffar sary, Ahmed fatma (2022). Ginger oil alleviates sero-biochemical and histopathological changes in pancreatic and liver tissues of diabetic induced rats. Assiut Veterinary Medical Journal.

[38] Lee J.C., Smith S.B., Watada H., Lin J., Scheel D., Wang J. (2001). Regulation of the pancreatic pro-endocrine gene neurogenin3. Diabetes.

[39] Al-Adsani A.M., Al-Otaibi A.N., Barhoush S.A., Al-Qattan K.K., Al-Bustan S.A (2022). Expression profiling of Pdx1, Ngn3, and MafA in the liver and pancreas of recovering streptozotocin-induced diabetic rats. Genes (Basel).

[40] Al‐Adsani A.M., Al‐Qattan K.K., Barhoush S.A., Abbood M.S., Al‐Bustan S.A (2024). Garlic extract promotes pancreatic islet neogenesis through α‐to‐β‐cell transdifferentiation and normalizes glucose homeostasis in diabetic rats. Mol. Nutr. Food Res..

[41] Bahrami G., Sajadimajd S., Mohammadi B., Hatami R., Miraghaee S., Keshavarzi S. (2020). Anti-diabetic effect of a novel oligosaccharide isolated from rosa canina via modulation of DNA methylation in streptozotocin-diabetic rats. Daru.

[42] Wang C., Yao J., Ju L., Wen X., Shu L (2020). Puerarin ameliorates hyperglycemia in HFD diabetic mice by promoting β-cell neogenesis via GLP-1R signaling activation. Phytomedicine.

[43] Matsuoka T., Zhao L., Artner I., Jarrett H.W., Friedman D., Means A. (2003). Members of the large Maf transcription family regulate insulin gene transcription in islet beta cells. Mol. Cell. Biol..

[44] Bsharat S., Monni E., Singh T., Johansson J.K., Achanta K., Bertonnier-Brouty L. (2023). MafB-dependent neurotransmitter signaling promotes β cell migration in the developing pancreas. Development.

[45] Holter M.M., Saikia M., Cummings B.P (2022). Alpha-cell paracrine signaling in the regulation of beta-cell insulin secretion. Front. Endocrinol. (Lausanne).

[46] Wei T., Cui X., Jiang Y., Wang K., Wang D., Li F. (2023). Glucagon acting at the GLP-1 receptor contributes to β-cell regeneration induced by glucagon receptor antagonism in diabetic mice. Diabetes.

[47] Moede T., Leibiger I.B., Berggren P.O (2020). Alpha cell regulation of beta cell function. Diabetologia.

[48] Kilimnik G., Kim A., Steiner D.F., Friedman T.C., Hara M (2010). Intraislet production of GLP-1 by activation of prohormone convertase 1/3 in pancreatic α-cells in mouse models of ß-cell regeneration. Islets.

[49] Zhang Z., Hu Y., Xu N., Zhou W., Yang L., Chen R. (2019). A new way for beta cell neogenesis: transdifferentiation from alpha cells induced by glucagon-like peptide 1. J. Diabetes Res..

[50] Wei R., Cui X., Feng J., Gu L., Lang S., Wei T. (2020). Dapagliflozin promotes beta cell regeneration by inducing pancreatic endocrine cell phenotype conversion in type 2 diabetic mice. Metab. Clin. Exp..

[51] Pan F.C., Wright C (2011). Pancreas organogenesis: from bud to plexus to gland. Dev. Dyn..

[52] Okuno M., Minami K., Okumachi A., Miyawaki K., Yokoi N., Toyokuni S. (2007). Generation of insulin-secreting cells from pancreatic acinar cells of animal models of type 1 diabetes. Am. J. Physiol. Endocrinol. Metab..

[53] Johansson K.A., Dursun U., Jordan N., Gu G., Beermann F., Gradwohl G. (2007). Temporal control of neurogenin3 activity in pancreas progenitors reveals competence windows for the generation of different endocrine cell types. Dev. Cell.

[54] Yin D., Tao J., Lee D.D., Shen J., Hara M., Lopez J. (2006). Recovery of islet beta-cell function in streptozotocin- induced diabetic mice: an indirect role for the spleen. Diabetes.

[55] Sarnobat D., Moffett R.C., Ma J., Flatt P.R., McClenaghan N.H., Tarasov A.I (2023). Taurine rescues pancreatic β-cell stress by stimulating α-cell transdifferentiation. Biofactors.

[56] Singh R., Gholipourmalekabadi M., Shafikhani S.H (2024). Animal models for type 1 and type 2 diabetes: advantages and limitations. Front. Endocrinol. (Lausanne).

[57] Furman B.L (2021). Streptozotocin-induced diabetic models in mice and rats. Curr. Protoc..

[58] National research council (US) committee for the update of the guide for the care and use of laboratory animals (2011). Guide for the Care and Use of Laboratory Animals.

[59] Al-Adsani A.M., Barhoush S.A., Bastaki N.K., Al-Bustan S.A., Al-Qattan K.K (2022). Comparing and optimizing RNA extraction from the pancreas of diabetic and healthy rats for gene expression analyses. Genes (Basel).

